# Sonic hedgehog signaling in astrocytes

**DOI:** 10.1007/s00018-020-03668-8

**Published:** 2020-10-20

**Authors:** Steven A. Hill, Marissa Fu, A. Denise R. Garcia

**Affiliations:** 1 Department of Biology, Drexel University, Philadelphia, PA 19104 USA; 2 Department of Neurobiology and Anatomy, Drexel University College of Medicine, Philadelphia, PA 19129 USA

**Keywords:** Astrocyte, Sonic hedgehog, Gli1, Glia, Neuron-astrocyte communication

## Abstract

Astrocytes are complex cells that perform a broad array of essential functions in the healthy and injured nervous system. The recognition that these cells are integral components of various processes, including synapse formation, modulation of synaptic activity, and response to injury, underscores the need to identify the molecular signaling programs orchestrating these diverse functional properties. Emerging studies have identified the Sonic hedgehog (Shh) signaling pathway as an essential regulator of the molecular identity and functional properties of astrocytes. Well established as a powerful regulator of diverse neurodevelopmental processes in the embryonic nervous system, its functional significance in astrocytes is only beginning to be revealed. Notably, Shh signaling is active only in discrete subpopulations of astrocytes distributed throughout the brain, a feature that has potential to yield novel insights into functional specialization of astrocytes. Here, we discuss Shh signaling and emerging data that point to essential roles for this pleiotropic signaling pathway in regulating various functional properties of astrocytes in the healthy and injured brain.

## Introduction

Astrocytes are the most abundant glial cells in the brain and are vital for normal brain function. Astrocytes are no longer relegated to the sidelines as monolithic “support cells”, and crosstalk between astrocytes and neurons is now known to be required for a number of important processes [1]. Astrocytes are required for synapses to form between neurons, and a number of studies have identified astrocyte-derived molecules that are required for the formation and function of individual synapses [2]. These include both astrocyte-secreted molecules, such as SPARC, hevin, thrombospondins, and chordin-like 1, whose presence regulates the insertion of specific receptors into developing synapses, and direct astrocyte–neuron contact via neuroligin/neurexin linkages, which also regulates astrocyte morphogenesis [3–7]. In addition to their roles in synapse formation and maturation, astrocytes are also vital for neuronal circuit function through their regulation of the extracellular ionic environment. Disruptions in astrocytic proteins, such as the inward rectifying potassium channel K_ir_4.1, produce circuit abnormalities throughout the CNS, including in the lateral habenula, striatum, and spinal cord, which are implicated in neurological diseases, such as depression, Huntington’s disease, and amyotrophic lateral sclerosis, respectively [8–10]. There is also evidence that astrocytes sense and respond to neurons via calcium-mediated mechanisms that result in secreted neuromodulators to regulate circuit activity [11, 12]. These are, but a few examples of the myriad functions of astrocytes that have been recently elucidated. However, while there have been a number of important insights into the functional diversity of astrocytes, the particular molecular profiles that govern this diversity remain elusive [13–18].

Insight into the unique molecular profiles of different astrocyte populations has been achieved from a number of recent studies, and there is now substantial evidence that astrocytes are a diverse and heterogeneous cell type in the brain. Although early transcriptomic studies identified genes shared in all astrocytes throughout the brain or changes in gene expression in a particular region over time, the recent studies have identified transcriptional differences between astrocytes of different regions and point to inherent heterogeneity between different astrocyte populations [19, 20]. Unique gene profiles have been identified between astrocytes of the cortex, striatum, brainstem, and hippocampus [21–23]. In addition to region-specific differences in gene expression, astrocyte diversity has also been shown within single brain regions. At least 5 different astrocyte populations were identified in the cortex and hippocampus using single-cell RNA sequencing, and transcriptional diversity between astrocytes in the cortex suggest that astrocytes exhibit a cortical layering pattern defined by distinct gene-expression profiles [17, 18]. Together, these studies provide evidence for astrocyte diversity both between and within regions and highlight the need to identify specific molecular signaling programs to identify the functional significance conferred by this remarkable transcriptional diversity.

The Sonic hedgehog (Shh) signaling pathway represents an exciting opportunity to interrogate molecularly defined populations of astrocytes and investigate their unique functional properties. Although best understood for its essential roles in patterning the nervous system during development, in the adult brain, Shh activity is found in discrete populations of mature astrocytes [24]. Cells actively transducing SHH are identified by the expression of the transcription factor GLI1. Interestingly, SHH is produced by neurons, suggesting it mediates neuron–astrocyte communication in the adult brain [24, 25]. The recent studies have demonstrated important roles for Shh signaling in multiple processes, including astrocyte modulation of neuronal activity and defining specific molecular signatures, suggesting that Shh regulates key functional properties of astrocytes [26, 27]. Essential features of Shh signaling in astrocytes, including the neuronal source of ligand and its distribution in discrete subpopulations of astrocytes, have the potential to provide novel insight into many open questions in astrocyte biology, such as astrocyte heterogeneity and astrocyte-neuron interactions. Here, we discuss the emerging body of literature on the diverse roles of Shh signaling in astrocyte function.

## Overview of Sonic hedgehog signaling

Shh signaling is best understood for its diverse roles during embryonic and early postnatal development where it mediates a broad range of developmental processes [28, 29]. SHH exhibits a ventral–dorsal gradient in the developing neural tube in which SHH released from the notochord and ventral floor plate specifies the fate of neural precursor cells in a concentration-dependent manner. Both neurons and oligodendrocytes are specified through SHH-dependent regulation of specific transcription factors, including *Nkx2.2* and *Olig2,* whose precise expression defines progenitor cell domains and identity [30–32]. In the cerebellum, Shh signaling regulates both the foliation and proliferation of cerebellar neural precursor cells, and induces granule cell progenitor division and migration from the external germinal layer to the inner granule layer [33–35]. In addition to its mitogenic roles, Shh signaling also plays a critical role in axon pathfinding, where it acts as both a chemoattractant and repellant for commissural axons in the developing spinal cord [36–38]. Likewise, in the developing retina, SHH guides proper wiring of retinal ganglion axons through the optic chiasm [39]. Thus, Shh signaling exerts powerful influence over diverse processes that are central to establishing the organization of the CNS.

**Fig. 1:**
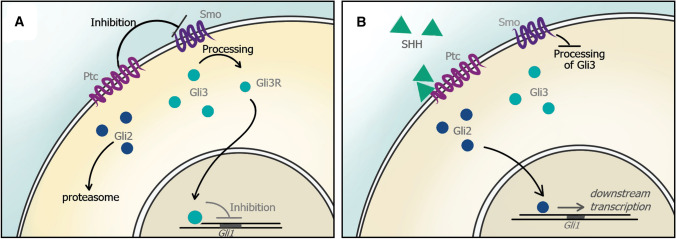
The Shh pathway. **a** In the absence of SHH, SMO activity is inhibited by PTC. Cytosolic GLI3 is processed as a repressor (GLI3R) and inhibits transcription of Shh target genes. **b** Binding of SHH to PTC alleviates inhibition of SMO, and GLI2 is proteolytically processed as an activator and is translocated to the nucleus, activating downstream transcription of SHH target genes, including GLI1

A number of excellent reviews have covered the molecular mechanisms of Shh signaling but we will provide a brief overview here [29, 40, 41]. Shh signaling is transduced and orchestrated within the primary cilium, a small microtubule-rich protrusion present on all cells, including astrocytes [42, 43]. The canonical pathway includes the twelve-pass transmembrane receptor, patched1 (PTCH1), its obligate seven-pass transmembrane co-receptor, smoothened (SMO), and its effector proteins, the zinc finger transcription factors known as GLIs (glioblastoma gene products 1–3; Fig. 1). In the absence of SHH, PTCH1 inhibits the activity of SMO through mechanisms that are not yet well understood. GLI3 is processed into its repressor form (GLI3R), which inhibits transcriptional activation of Shh pathway genes [44–49]. Binding of SHH to PTCH1 relieves inhibition of SMO triggering signal transduction within the cell [50]. Upon SMO activation, GLI2 acts as the primary transcriptional activator of the pathway and initiates transcription of downstream Shh target genes, including *Gli1* [49, 51]. GLI1 is a transcriptional activator, and its presence is indicative of active Shh activity within the cell [44, 52–54]. Importantly, SHH is absolutely required for GLI1 expression, as SHH mutant embryos fail to transcribe *Gli1* [55]. Transcriptional activation of *Gli1* is therefore a reliable read-out of active Shh signaling within the cell.

## Shh signaling in astrocytes

**Fig. 2:**
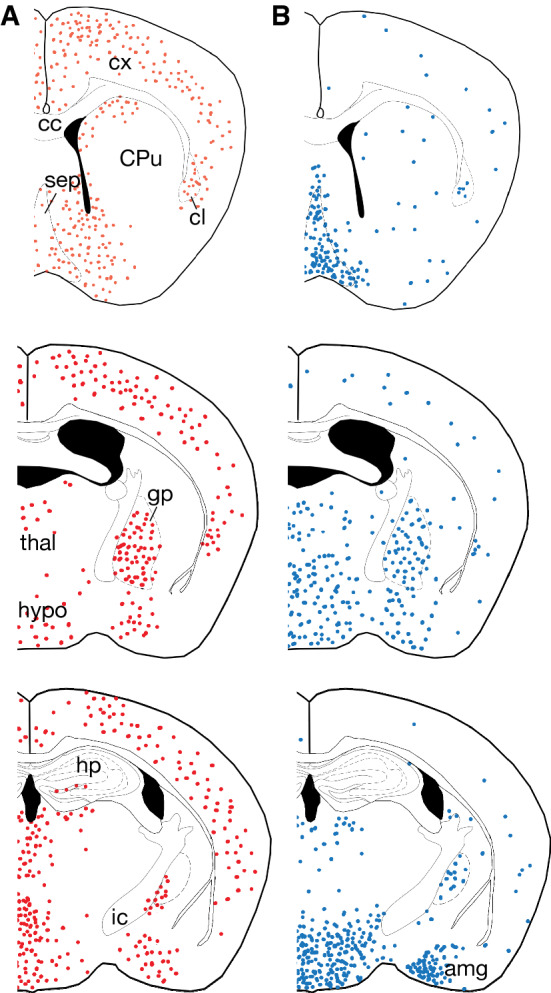
Distribution of *Shh* and *Gli1*-expressing cells in the adult forebrain. **a** Schematics depicting the distribution of astrocytes expressing *Gli1* (red) across three levels of the anterior/posterior axis. Note that these cells are found in high abundance in various regions including the hypothalamus, globus pallidus and cortex, but are noticeably absent in other regions, such as the white matter and hippocampus. **b** The distribution of *Shh*-expressing neurons (blue), as observed from genetic labeling experiments. *cx* cortex, *hypo* hypothalamus, *gp* globus pallidus, *cc* corpus callosum, *CPu* caudate putamen, *hp* hippocampus, *amg* amygdala

Early studies identified the persistence of Shh signaling throughout the adult mammalian CNS using in situ hybridization. *Ptch1*, *Smo*, and *Shh* transcripts were found in the spinal cord and various brain regions, including the hypothalamus, cortex, and the cerebellum [56, 57]. One limitation of the in situ hybridization studies is the inability to identify cell type. However, the development of molecular genetic tools to label cells with *Gli1* activity identified astrocytes as the predominant cell type actively transducing Shh signaling in the adult mouse brain [24]. Astrocytes express *Ptch1*, *Smo*, *Gli2,* and *Gli3*, demonstrating that they possess the key machinery to transduce SHH [19]. Notably, transduction of SHH is restricted to regionally defined subpopulations, suggesting that these cells may possess specific molecular signatures and functional properties that distinguish them from other astrocytes. Astrocytes that express *Gli1* are distributed throughout the forebrain, including in the hypothalamus, thalamus, globus pallidus, and deep layers of the cortex. In contrast, the striatum and white matter tracts are largely devoid of *Gli1*-expressing astrocytes, as is the hippocampus, with the exception of adult neural stem cells in the dentate gyrus [58] (Fig. 2). Notably, the proportion of astrocytes exhibiting active Shh signaling in different brain regions varies, with ventral regions, such as the hypothalamus harboring a larger fraction of *Gli1*-expressing astrocytes than the cortex [24]. This distribution suggests that astrocytes may possess functional specialization both between disparate brain regions, as well as within a given region, that may be regulated by Shh signaling.

**Fig. 3:**
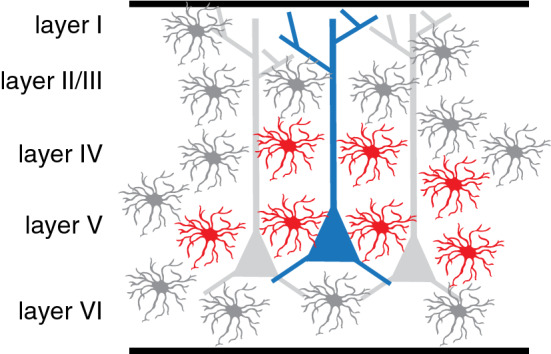
Laminar distribution of *Shh* and *Gli1*-expressing cells in the cortex. In the somatosensory cortex, astrocytes expressing *Gli1* (red) are localized predominantly in layers IV and V. Neurons expressing *Shh* (blue) are found largely in layer V

Interestingly, while *Gli1* is expressed primarily in astrocytes, *Shh* in the healthy, adult mammalian brain is expressed by neurons, suggesting that Shh signaling mediates neuron-astrocyte communication [24, 26, 59, 60]. As in the developing embryo, SHH in the adult brain is found predominantly in ventral regions, such as the hypothalamus, which harbors a large number of *Shh*-expressing neurons and *Gli1*-expressing astrocytes (Fig. 2) [24]. *Shh* + neurons are also found in the cortex, where genetic marking studies show that they correspond mostly to pyramidal cells [25]. In the somatosensory cortex, there is an apparent laminar distribution such that *Shh* + neurons are found predominantly in layer V while astrocytes expressing *Gli1* are found primarily in layers IV and V [27] (Fig. 3). The functional significance of this precise distribution pattern is not well understood, but a recent study suggests that Shh-dependent local interactions between neurons and astrocytes mediates synaptic function and organization of layer V neurons, as discussed further below [27]. Further studies should explore whether this distribution is specific to the somatosensory cortex or whether this is found in other cortical regions. More recently, it has also been shown that mature oligodendrocytes may also be a source of SHH in the adult brain [61]. The extent to which transduction of Shh signaling in individual astrocytes or specific astrocyte populations is due to neuronal or oligodendrocyte sources of SHH requires further study.

## The functional significance of Shh signaling in astrocytes

The gene expression programs regulated by Shh signaling in neural precursor cells are well characterized and include programs associated with cell fate and proliferation [62]. In contrast, the Shh-dependent gene expression programs in differentiated astrocytes of the mature brain are only beginning to be elucidated. Growing evidence, however, points to an essential role in modulating synaptic function and neuronal activity. In the cortex, Shh signaling between layer V neurons and neighboring astrocytes is required for the refinement of synapses during neural circuit development. Conditional mutants in which *Smo* is selectively deleted in astrocytes exhibit impairments in structural plasticity and organization and concomitant reduction in expression of K_ir_4.1 [27]. In these mutants, layer V cortical neurons show significant changes in spine density and long-term synapse plasticity. These structural phenotypes are accompanied by heightened neuronal excitability, suggesting that Shh signaling initiates reciprocal interactions between neurons and astrocytes that exert functional and structural regulation of developing neural circuits. Importantly, these phenotypes are not observed in mutants lacking *Smo* in neurons, highlighting the requirement for Shh signaling in astrocytes on synapse activity and function. Interestingly, Shh signaling between Layer V and Layer II/III neurons mediates the precise wiring between these cells during postnatal cortical development [25]. Because neurons do not express *Gli1*, this suggests that these actions are GLI-independent and mediated by non-canonical Shh signaling [63]. This suggests that Shh signaling exerts essential regulation of synaptic connectivity, through both heterotypic and homotypic cellular interactions.

In the cerebellum, Shh signaling drives expression of genes that confer specific molecular identities to different classes of astroglial cells [26]. Bergmann glia are localized to the Purkinje cell layer and extend their processes into the molecular layer toward the apical surface of the cerebellar cortex, where they associate with the dendrites of Purkinje neurons. They exhibit robust expression of *Ptch1*, and *Smo* transcripts and transduce SHH derived from neighboring Purkinje cells [26]. Genetic deletion experiments demonstrate that Shh signaling regulates expression of Kir4.1 in Bergmann glia, as observed in the cortex [26, 27]. Interestingly, Shh activity also regulates expression of AMPA receptor subunits, GluA1 and GluA4 in Bergmann glia, further supporting the role of Shh signaling in astrocyte modulation of synaptic activity [26]. Velate astrocytes, on the other hand, reside in the granule cell layer, below Purkinje cells, and receive lower levels of SHH. In contrast to Bergmann glia, velate astrocytes express low levels of GluA1 and GluA4. However constitutive activation of Shh activity in velate astrocytes upregulates expression of these proteins [26]. These observations demonstrate that Shh signaling is both necessary and sufficient to regulate molecular characteristics of distinct astrocyte classes.

In both the cortex and cerebellum, Shh signaling regulates expression of genes in astrocytes that are required to mediate their fundamental role as essential regulators of the extracellular synaptic environment. Specifically, Shh signaling affects the ability of astrocytes to clear K + and glutamate, both of which must be tightly regulated to ensure appropriate synaptic activity and neuronal survival [64–67]. In addition to its role in mediating astrocyte modulation of synaptic activity, there is also evidence that Shh signaling also influences the ability of astrocytes to communicate with neurons via gliotransmission [68–70]. Application of SHH on cultured astrocytes initiates an intracellular calcium response and subsequent release of both ATP and glutamate, and blocking Shh activity or chelating intracellular calcium inhibited their release [68, 70]. Taken together, these demonstrate that SHH influences astrocyte-synapse interactions in diverse ways, and point to Shh signaling in astrocytes as an important mediator of bidirectional communication between neurons and astrocytes.

## Shh signaling in reactive astrocytes

A growing body of evidence shows that Shh signaling exerts neuroprotective influence on the injury microenvironment by limiting inflammation. Shh activity lowers permeability of the blood brain barrier (BBB), restricting entry of peripheral proteins and blood-borne macrophages into the CNS parenchyma that can elicit toxic inflammatory signaling [71, 72]. Importantly, this effect has been observed in diverse injury environments, including spinal cord injury contusion, ischemia, and cortical stab wound, suggesting a central role of the pathway in neuroinflammation [73–75]. Astrocytes are key cellular mediators of the anti-inflammatory action of Shh signaling. Application of the SHH agonist, SAG, limits accumulation of leukocytes in CNS parenchyma following cortical stab wound. SAG acts directly on SMO, the obligatory co-receptor that activates the pathway. Genetic deletion of *Smo* selectively in astrocytes abolishes the effect of SAG on leukocyte accumulation [75]. Notably, despite the powerful anti-inflammatory activity of the pathway, these mutants show no signs of a weakened BBB in the absence of injury, suggesting that Shh activity may play a role in repressing injury-induced cytokine production by reactive astrocytes [75]. In addition to its action on astrocytes, Shh signaling has also been shown to act on endothelial cells, regulating expression of tight junction proteins. Shh activity promotes expression of the junctional proteins occludin and claudin-5 in vitro and in vivo, promoting BBB integrity [71]. Conversely, permeability assays in cultured endothelial cells show that the pro-inflammatory cytokine, IL-1β, suppresses *Shh* and downregulates expression of tight junction proteins [76]. This suggests that inflammation suppresses Shh signaling. Consistent with this, genetic labeling studies show that *Gli1* expression is lost in reactive astrocytes during the acute stages of injury, when inflammation is high, but is restored to baseline levels by 14 days after the insult, as inflammation is resolved [75, 77]. This is accompanied by a concomitant reduction, and subsequent restoration, of *Shh* gene expression [75]. Taken together, these studies point to Shh signaling as a potential target for mitigating injury-induced neurotoxic inflammation.

Following virtually all types of insults, astrocytes undergo pronounced changes in physiology, morphology, and gene expression, collectively referred to as reactive astrogliosis [3, 56, 57]. In its most severe form, astrogliosis includes proliferation of reactive astrocytes [72, 80]. Consistent with its role as a key regulator of proliferation in neural precursor cells, Shh signaling promotes proliferation of cells isolated from the cortex following an acute invasive injury [81]. Cultures prepared from cortical stab wound tissue generate reactive astrocyte-derived neurospheres in a SHH-dependent manner [81]. Application of SHH or its agonist SAG increase neurosphere formation whereas the SHH inhibitor, cyclopamine, blocks neurosphere formation, demonstrating that Shh activation is necessary and sufficient to stimulate proliferation in vitro [81]. However genetic inactivation studies demonstrate that reactive astrocyte proliferation in vivo occurs independently of Shh signaling [75]. Conditional mutants lacking *Smo* in astrocytes show no difference in proliferating cells at the lesion site following a cortical stab, despite reduced proliferation of GFAP-expressing adult neural stem cells in both the dentate gyrus of the hippocampus and in the subventricular zone [75, 82–84]. However as discussed above, this study also demonstrated a dramatic loss of Shh activity in reactive astrocytes during the first week following insult, the time during which reactive astrocytes proliferate [72]. Thus, although endogenous Shh activity is suppressed during the initial acute stages of injury, the observations from the neurosphere assay suggest that activation of the pathway in vivo can promote proliferation of reactive astrocytes and other proliferating cell populations. Indeed, across various injury models, including spinal cord injury, ischemia, and demyelination, Shh signaling promotes proliferation of neural precursor cells, including oligodendrocyte progenitor cells as well as adult neural stem cells in the lateral ventricles or dentate gyrus [73, 74, 77, 85–88].

While these studies suggest that Shh may be a promising target for mitigating CNS damage following various types of trauma, conflicting reports on the direction of endogenous Shh activity following injury, as well as the precise cellular sources and targets of the pathway, leave open the question of the actions of Shh signaling in vivo in the injury environment. Whereas early studies reported an increase in Shh activity following injury, and identified astrocytes as cellular sources of SHH, later studies using genetic labeling approaches demonstrate a loss of Shh activity. Using transgenic mice carrying a Gli-luciferase reporter, an increase in bioluminescence was observed up to 1 week after both a cortical stab wound and kainic-acid induced lesion, suggesting an increase in Shh activity following both an acute invasive injury model and acute neurodegeneration [85, 89]. Similar observations were reported in ischemic models, in which middle cerebral artery occlusion increases expression of various components of the Shh pathway, as measured by qPCR or antibody labeling [74, 81, 90, 91]. Antibody labeling has shown that astrocytes are the source of SHH in several studies and injury models, whereas other studies report neurons and cerebrospinal fluid as the source of SHH [71, 81, 85, 89–91].

In more recent studies, the application of genetic labeling strategies to mark and identify cells expressing *Gli1* instead demonstrate loss of Shh activity immediately following the insult that persists for up to 2 weeks [75, 77]. Studies using double transgenic mice carrying the tamoxifen-inducible CreER at the *Gli1* locus and a Cre-dependent reporter (*Gli1*^*CreER/*+^*;R26R*) show that tamoxifen administered to mice within 3 days after a mild cortical contusion injury or stab injury show fewer or no *Gli1*-expressing astrocytes at the lesion site, compared to contralateral or uninjured controls [75, 77]. Chemical demyelination in *Gli1*^*CreER/*+^*;R26R* mice fed cuprizone for 6 weeks similarly show fewer labeled cells in the cortex when tamoxifen is administered in the fifth week, suggesting that the loss of *Gli1* activity persists in environments experiencing sustained injury [88]. These observations are supported by direct measurements of *Shh* by qPCR showing reductions in gene expression following stab injury or cuprizone-mediated demyelination [75, 92]. While different injury models produce distinct microenvironments that trigger differential gene expression programs [78, 79], the observations that *Gli1* expression is both increased and decreased following a cortical stab wound suggest that injury type alone cannot account for these conflicting observations. Notably, a similar injury-induced reduction in Shh activity is found in lung tissues where *Shh* is found in epithelial cells and *Gli1* is expressed in adjacent mesenchymal cells [93]. Using Cre-mediated labeling of *Gli1*-expressing cells, Peng et al. [93] observed that chemical injury to the lung produces a transient downregulation of Shh signaling that is associated with epithelial expansion and regeneration. As in the brain, Shh signaling is restored following resolution of the injury, supporting an essential role for context-dependent cues that regulate activity of the pathway.

These conflicting reports of the direction and sources of SHH after injury, together with the growing evidence that Shh signaling exerts powerful anti-inflammatory actions in the injured environment, underscore the need for further studies. Nevertheless, whether reactive astrocytes are the source or the effectors of Shh signaling, these studies point to astrocytes as key cellular mediators in the neuroprotective actions of Shh activity. Because SHH is a secreted protein, identifying the precise cellular source responsible for production of the protein can be difficult using antibody labeling in which localization of the antibody on cell surfaces cannot be ruled out. Genetic labeling experiments using transgenic mice carrying inducible Cre-dependent reporters would permit direct readouts of transcriptional activity within an individual cell. Coupled with double labeling for cell-type specific markers, such an approach would provide a reliable and robust strategy for resolving these conflicting observations, and move towards a better understanding of the precise role and mechanisms of action of Shh signaling in injury.

## Outstanding questions

### How is SHH released?

The mechanism by which SHH is released from cells in the postnatal and adult CNS is not well understood. In the embryonic CNS, SHH is secreted from the notochord and floor plate, and diffuses dorsally across the ventral neural tube where interactions with extracellular matrix proteins, such as heparin sulfate proteoglycan (HSPG), help establish a gradient [41, 94]. Such concentration-dependent transduction of Shh signaling in astrocytes has not been shown. However, examination of the relative distributions of *Shh*-expressing neurons and *Gli1*-expressing astrocytes suggests that passive diffusion of ligand may mediate local interactions between neighboring neurons and astrocytes. In the cerebellum, SHH expressed by Purkinje neurons is transduced by neighboring Bergman glial cells [26]. Likewise, regions, such as the septum, hypothalamus and globus pallidus possess a high proportion of *Gli1*-expressing astrocytes that are associated with local populations of *Shh*-expressing neurons [24] (Fig. 2). Indeed, genetic marking experiments in adult *Gli1CreER* mice show that approximately 80% of astrocytes in the hypothalamus express *Gli1*, suggesting that these cells are responding to local availability of high amounts of SHH [24].

In contrast, the cortex displays an apparent mismatch between the relative numbers of *Gli1*-expressing astrocytes and *Shh*-expressing neurons that are observed by genetic marking [24] (Fig. 2). Despite the considerable number of astrocytes in the adult cortex that express *Gli1*, the number of *Shh* + neurons identified by genetic labeling experiments is relatively small. One possibility is that additional sources of SHH are available from cells that fail to undergo Cre-mediated recombination in genetic labeling experiments. Indeed, both astrocytes and oligodendrocytes have been shown to express SHH by antibody labeling, though this has not been observed in genetic labeling experiments [61, 81, 89, 91]. *Gli1* expression in cortical astrocytes may occur independent of SHH. However loss of one copy of *Shh* leads to a significant reduction in *Gli1* expression arguing against this possibility [24]. Alternatively, an intriguing possibility is that astrocytes may also be responding to SHH from distal neurons projecting to the cortex. There is evidence that SHH is transported axonally and is released at synapses [95–97]. SHH has been observed in retinal ganglion cell (RGCs) axons and in axons originating in ventral forebrain neurons that project to the SVZ [59, 95, 98–100]. In the basal ganglia, dopaminergic neurons in the substantia nigra express SHH that is required for normal physiology and survival of GABAergic neurons in the striatum [101]. Moreover, SHH is found within vesicles in presynaptic terminals of neurons, and in vitro studies have demonstrated SNAREs-dependent (soluble n-ethylmaleimide-sensitive fusion protein attachment protein-receptors) release of SHH following high frequency stimulation [96, 102]. Uncovering whether astrocytes transduce SHH from neighboring cells, or whether astrocytes can respond to SHH from regionally distant cells is an important question that should be explored in future experiments.

### Does SHH confer functional specialization to specific astrocyte populations?

The idea that astrocytes represent a heterogeneous population of cells with distinct molecular signatures and functional specialization has emerged as an exciting topic of intense interest over the past decade [14, 15, 103, 104]. Though classically regarded as a uniform cell population, advances in our understanding of the regional and functional diversity of astrocytes are re-shaping this view [18, 21, 22, 105, 106]. Astrocytes across brain regions exhibit diversity in calcium activity, synaptic coverage, and expression of channels and transporters that regulate synaptic activity [13, 14, 16, 107]. The observation that transduction of SHH is restricted to discrete subpopulations of astrocytes, distributed in region-specific ways, presents an opportunity to discover novel insights into astrocyte heterogeneity. One possibility is that SHH regulates gene expression programs that confer specific molecular and functional identities to specific astrocyte populations, consistent with its role in cell specification of uncommitted neural precursor cells during embryonic development. Indeed, differential transduction of Shh between Bergmann glia and velate astrocytes in the cerebellum drives the unique molecular profiles of these two astroglial cells. It would be interesting to examine whether all astrocytes expressing *Gli1* throughout the brain share the same molecular signatures. Alternatively, Shh signaling may cooperate with local environmental cues to facilitate context-dependent gene expression, enabling astrocytes to perform region-specific functions. Recent evidence suggests that astrocytes in different brain regions exhibit vast transcriptomic, proteomic, and functional differences [22]. In this framework, Shh signaling functions less as a driver of specific cellular identity, but rather as a powerful tool with which the nervous system can instruct specific astrocyte populations to meet local needs. Understanding how astrocytes interpret SHH in different regions will facilitate a better understanding of astrocyte functional specialization.

### Does Shh signaling have a role in astrocyte development?

While the role of Shh signaling in oligodendrocyte development is well-established, considerably less is known about its role in astrocyte development. Astrocyte production occurs primarily during the first two postnatal weeks [108]. They are derived from radial glia and progenitor cells residing in the subventricular zone, as well as from local proliferation of differentiated cells [109–111]. In the developing optic nerve, SHH released from retinal ganglion cell axons regulates proliferation of astrocyte precursors [98]. Because *Gli1* expression is not found in white matter tracts in the adult forebrain, this suggests that Shh signaling in astrocyte precursors is developmentally regulated and that activity of the pathway in precursor populations does not predict activity in mature cells [24]. In the mature cortex, astrocytes expressing *Gli1* are found predominantly in deep layers. Whether Shh signaling plays a role in the production of these, or other *Gli1*-expressing astrocytes, remains an open question. Loss of Shh activity in postnatal glial progenitor cells does not impair the total number of cortical astrocytes in the mature brain, suggesting that their production does not require Shh signaling [24]. Nevertheless, expression of K_ir_4.1 is dramatically reduced in these conditional mutants, suggesting that Shh activity is important for gene expression programs associated with astrocyte function, but not their production [27]. Future studies employing fate mapping and intersectional genetics approaches would be a powerful strategy for elucidating the relationship between Shh signaling and astrocyte development.

## Conclusions and future perspectives

There has been a remarkable paradigm shift in our understanding of the vital roles astrocytes play in CNS function. No longer relegated to passive, functionally homogeneous cells, astrocytes have gained recognition as a complex class of cells with diverse roles in a broad range of CNS functions. The features that characterize Shh signaling in astrocytes, including the selective activation in specific subpopulations and the neuronal source of the initiating signal, present exciting opportunities to interrogate broad questions about astrocyte biology including molecular identity and functional specialization of these cells, as well as reciprocal interactions between astrocytes and neurons. Given the potent ability of Shh to regulate gene expression during development, it is plausible that Shh activity regulates gene expression programs that confer specific functional specialization onto a molecularly distinct class of astrocytes. Consequently, astrocytes expressing *Gli1* may be markedly different from those that do not. Alternatively, Shh signaling may be dynamic, with *Gli1* expression reflecting Shh activity that is regulated by local environmental cues. Indeed, there is evidence that neuronal activity stimulates release of SHH, and this may be used by neurons to recruit nearby astrocytes to regulate the extracellular environment during times of high activity [102]. Further studies elucidating the roles of Shh signaling in astrocytes in healthy and pathological states hold promise to yield novel insight and advancements in our understanding of astrocyte function, development, and heterogeneity.
